# Endovascular Treatment of Acute Ischemic Stroke With the Penumbra System in Routine Practice: COMPLETE Registry Results

**DOI:** 10.1161/STROKEAHA.121.034268

**Published:** 2021-09-22

**Authors:** Osama O. Zaidat, Johanna T. Fifi, Ashish Nanda, Benjamin Atchie, Keith Woodward, Arnd Doerfler, Alejandro Tomasello, Wondwossen Tekle, Inder Paul Singh, Charles Matouk, Jörg Thalwitzer, Tomasz Jargiełło, Dmitry Skrypnik, Oliver Beuing, Jérôme Berge, Jeffrey M. Katz, Alessandra Biondi, David Bonovich, Sunil A. Sheth, Albert J. Yoo, Ameer E. Hassan

**Affiliations:** Mercy Health St. Vincent Medical Center, Toledo, OH (O.O.Z.).; Icahn School of Medicine at Mount Sinai, New York, NY (J.T.F.).; SSM St. Clare Healthcare, Fenton, MO (A.N.).; RIA Neurovascular, Englewood, CO (B.A.).; Fort Sanders Regional Medical Center, Knoxville, TN (K.W.).; Universitätsklinikum Erlangen, Germany (A.D.).; Hospital Universitari Vall d’Hebron, Barcelona, Spain (A.T.).; University of Texas Rio Grande Valley - Valley Baptist Health System, Harlingen (W.T.).; Mount Sinai Health System, New York, NY (I.P.S.).; Yale New Haven Hospital, New Haven, CT (C.M.).; Klinikum Chemnitz GmbH, Chemnitz, Germany (J.T.).; Samodzielny Publiczny Szpital Kliniczny nr 4, Lublin, Poland (T.J.).; Moscow City Clinical Hospital named after Davydovsky, Russia (D.S.).; Universitätsklinikum Magdeburg, Germany (O.B.).; CHU de Bordeaux- Hôpital Pellegrin, Bordeaux, France (J.B.).; North Shore University Hospital, Northwell Health, Manhasset, NY (J.M.K.).; J Minjoz University Hospital, Besançon, France (A.B.).; Eden Medical Center, Castro Valley, CA (D.B.).; Department of Neurology, UTHealth McGovern Medical School, Houston, TX (S.A.S.).; Texas Stroke Institute, Dallas-Fort Worth (A.J.Y.).; University of Texas Rio Grande Valley - Valley Baptist Medical Center, Harlingen (A.E.H.).

**Keywords:** cerebral infarction, ischemic stroke, mortality, reperfusion, thrombectomy

## Abstract

**Background and Purpose:**

The purpose of the COMPLETE (International Acute Ischemic Stroke Registry With the Penumbra System Aspiration Including the 3D Revascularization Device) registry was to evaluate the generalizability of the safety and efficacy of the Penumbra System (Penumbra, Inc, Alameda) in a real-world setting.

**Methods:**

COMPLETE was a global, prospective, postmarket, multicenter registry. Patients with large vessel occlusion–acute ischemic stroke who underwent mechanical thrombectomy using the Penumbra System with or without the 3D Revascularization Device as frontline approach were enrolled at 42 centers (29 United States, 13 Europe) from July 2018 to October 2019. Primary efficacy end points were successful postprocedure angiographic revascularization (modified Thrombolysis in Cerebral Infarction ≥2b) and 90-day functional outcome (modified Rankin Scale score 0–2). The primary safety end point was 90-day all-cause mortality. An imaging core lab determined modified Thrombolysis in Cerebral Infarction scores, Alberta Stroke Program Early CT Scores, clot location, and occurrence of intracranial hemorrhage at 24 hours. Independent medical reviewers adjudicated safety end points.

**Results:**

Six hundred fifty patients were enrolled (median age 70 years, 54.0% female, 49.2% given intravenous recombinant tissue-type plasminogen activator before thrombectomy). Rate of modified Thrombolysis in Cerebral Infarction 2b to 3 postprocedure was 87.8% (95% CI, 85.3%–90.4%). First pass and postprocedure rates of modified Thrombolysis in Cerebral Infarction 2c to 3 were 41.5% and 66.2%, respectively. At 90 days, 55.8% (95% CI, 51.9%–59.7%) had modified Rankin Scale score 0 to 2, and all-cause mortality was 15.5% (95% CI, 12.8%–18.3%).

**Conclusions:**

Using Penumbra System for frontline mechanical thrombectomy treatment of patients with large vessel occlusion–acute ischemic stroke in a real-world setting was associated with angiographic, clinical, and safety outcomes that were comparable to prior randomized clinical trials with stringent site and operator selection criteria.

**Registration:**

URL: https://www.clinicaltrials.gov; Unique identifier: NCT03464565.

## Abstract 2

Supplemental Digital Content is available in the text.

Mechanical thrombectomy (MT) is the current standard of care for large vessel occlusion–acute ischemic stroke (LVO-AIS) treatment.^1,2^ Results from multiple randomized controlled trials support the safety and efficacy of MT.^3–8^ This may be related to improved patient selection and newer generation thrombectomy devices.

The American Heart/American Stroke Association 2019 and the European Stroke Organization/European Society for Minimally Invasive Neurological Therapy 2019 Guidelines agree that high-quality evidence exists to support the use of MT plus best medical management over best medical management alone in patients with anterior circulation LVO-AIS with an Alberta Stroke Program Early CT Score (ASPECTS) ≥6 presenting within 6 hours of onset. Both also agree that MT is reasonable and should be considered for patients with ASPECTS<6, or posterior circulation occlusions, but acknowledge the lack of high-quality evidence available for these less explored populations.^1,2^

The Penumbra System (Penumbra Inc, Alameda) is an MT system specifically designed to remove thrombus using aspiration.^9–11^ Several design and catheter size iterations have been made since the initial commercial launch and the Penumbra System was used in several trials, including ADAPT FAST (A Direct Aspiration First Pass Technique for Acute Stroke Thrombectomy),^12^ ASTER (The Contact Aspiration vs Stent Retriever for Successful Revascularization),^13^ PROMISE (Prospective Multicenter Imaging Study for Evaluation of Chest Pain),^14^ the 3-dimensional (3D) Randomized Trial,^15^ and COMPASS (Cardiovascular Outcomes for People Using Anticoagulation Strategies)^16^—providing additional clinical data supporting the Penumbra System’s performance. Citing results of COMPASS,^16^ ASTER,^13^ and the 3D Randomized Trial,^15^ the American Heart/American Stroke Association guidelines endorse frontline aspiration thrombectomy as noninferior to stent retrievers.^2^ These trials had strict site selection criteria, enrollment criteria, and restricted patient population by anterior circulation LVO location,^13,14,16^ time from symptom onset,^13–16^ and eligibility-for or response-to intravenous thrombolytic therapy.^15^ Furthermore, several recent additions to the Penumbra System were not included in these prior trials.

This study, the COMPLETE (International Acute Ischemic Stroke Registry With the Penumbra System Aspiration Including the 3D Revascularization Device) Registry, evaluated the generalizability of the Penumbra System’s performance in a real-world setting.

## Methods

COMPLETE was a global, prospective, multicenter, single-arm, postmarket, registry assessing the performance of the Penumbra System in an LVO-AIS patient population. Forty-two sites (29 United States, 13 Europe) enrolled patients from July 2018 through October 2019, and 90-day follow-up was completed in January 2020. The data that support the findings of this study are available from the corresponding author upon reasonable request.

Inclusion criteria were patient age ≥18 years, prestroke modified Rankin Scale (mRS) score 0 to 1, patient experiencing AIS secondary to intracranial LVO who is eligible for MT using Penumbra System, planned frontline treatment with Penumbra System, and signed informed consent per Institutional Review Board/Ethics Committee.

Exclusion criteria were any comorbid disease or condition expected to compromise survival or ability to complete follow-up assessments through 90 days and currently participating in an investigational (drug, device, etc) clinical trial that will confound registry end points. Patients in observational, natural history, or epidemiological studies not involving intervention remained eligible.

Investigators used routine clinical evaluations to determine patient eligibility. Advanced imaging was not required but permissible per site standard of care. A screening and enrollment log of all MT eligible patients with LVO-AIS was maintained at each participating hospital with reason(s) for exclusion recorded. Patients were considered enrolled once informed consent was obtained and Penumbra System was inserted into the body. Participating hospitals entered data into the Oracle InForm electronic data capture system (Oracle, Austin, TX). To ensure data accuracy and protocol compliance, the sponsor implemented risk-based monitoring. The RECORD (Reporting of Studies Conducted Using Observational Routinely-Collected Data) statement checklist and study flow diagram are available in the Data Supplement.^17^

Frontline treatment modality (direct aspiration only or aspiration combined with a 3D Revascularization Device) was decided by the treating physician. Procedures were conducted in accordance with routine care. Available devices included the MAX, ACE, and JET reperfusion catheters; the 3D Revascularization Device; and the Pump MAX, and ENGINE aspiration sources. The catheter distal inner dimensions ranged from 0.035” (0.89 mm) to 0.072” (1.83 mm). The aspiration sources are designed to deliver and maintain a consistent vacuum (−29.2 in Hg or 98.9 kPa for ENGINE).

The following predefined cohorts represent different patient populations and were reported separately: cohort A (anterior LVO with ASPECTS≥6), cohort B (anterior LVO with ASPECTS<6), and cohort C (posterior LVO). All end point analyses were conducted in cohort A. Exploratory analyses were conducted in cohorts B and C.

### End Points

The primary efficacy end points were angiographic revascularization of the occluded target vessel at immediate postprocedure as defined by modified Thrombolysis in Cerebral Infarction (mTICI) score ≥2b and functional subject outcome at 90 days postprocedure defined as mRS score 0 to 2. mTICI 2b was defined as substantial reperfusion with distal branch filling of ≥50% of territory visualized. The primary safety end point was all-cause mortality at 90 days.

The secondary end points included incidence of device- and procedure-related serious adverse events (SAEs), occurrence of embolization in previously uninvolved or new territories (ENT) as seen on the final control angiogram at the end of procedure, occurrence of symptomatic intracranial hemorrhage (sICH) at 24 hours, and time to revascularization.

Efforts were made to reduce the number of patients lost to follow-up. At a minimum, this included 3 attempts to make contact via telephone or email, and if unsuccessful, a letter from the investigator was sent via certified mail or other traceable methods to the patient’s last known address. Patients were considered lost to follow-up if these efforts failed. Assessments of mRS were performed per site standard of care. Other end point information is presented based on imaging core lab or independent medical reviewer assessments.

### Study Committees

An independent imaging core lab reviewed pseudonymized angiography for mTICI scores, preprocedure CT for ASPECTS, computed tomography angiography for clot location, and 24-hour CT to assess hemorrhagic transformation using ECASS (European Cooperative Acute Stroke Study) classification.

Independent medical reviewers adjudicated all device-related SAEs, neurovascular procedure–related SAEs, sICH within 24 hours, events of neurological deterioration, and any deaths that occurred throughout the registry. Neurological deterioration was defined as a ≥4-point worsening of the National Institutes of Health Stroke Scale score from baseline, and sICH was defined as 24-hour evidence of an ECASS defined ICH associated with a ≥4-point worsening of the National Institutes of Health Stroke Scale score from baseline.

### Institutional Review Board/Ethics Committees

The study and informed consent process were approved by the Institutional Review Board/Ethics Committee for each participating center before registry initiation. Informed consent was obtained for all enrolled patients per local Institutional Review Board/Ethics Committee requirements. See Data Supplement for a complete list of all Institutional Review Board/Ethics Committee. The informed consent process was consistent with the applicable elements of EN ISO14155, clinical investigation of medical devices for human subjects—good clinical practice and 21 Code of Federal Regulations Part 50 and 54. In the United States, for emergent cases where the patient was unable to provide consent and a legally authorized representative was absent before the procedure, informed consent could be obtained within 2 calendar days. In Europe, informed consent was obtained for all cases before the procedure from the patient, next of kin, legally authorized representative, or 1 or 2 independent physician(s); if informed consent could not be obtained directly from the patient before procedure, it was obtained again before discharge either from the patient or other authorized persons per local Ethics Committee requirements.

### Statistical Analysis

Data were summarized using standard descriptive statistics (number of observations, mean, median, SD, and interquartile range for continuous variables, and counts and percentages for discrete variables). Statistical tests for continuous variables used the Wilcoxon Mann-Whitney rank-sum test and categorical variables were tested using Fisher exact test. Two-sided Exact Clopper-Pearson CI are presented. All statistical tests are 2-tailed with a significance level of 0.05. Sensitivity analyses were done using 3 imputation strategies for patients with missing primary efficacy end point data: poor outcome (mRS score 3–6), good outcome (mRS score 0–2), and multiple imputation. Poolability analysis with a hierarchical linear mixed model accounting for patient characteristics was performed. See Data Supplement for additional details. Analyses were conducted using SAS (SAS Institute, Cary, NC).

An estimated sample size of 650 was chosen for its ability to provide a sufficient level of precision to assess the Penumbra System and 3D Revascularization Device in the prespecified primary cohort A based on previously reported incidence rates. Approximately 500 patients (≈77% of enrollment) were anticipated to be in cohort A. Assuming a postprocedure angiographic success rate of 85% (425/500), the expected binomial 2-sided 95% CI was (81.9%–88.1%). Assuming 10% attrition and a 90-day mRS success rate of 45% (202/450), the expected binomial 2-sided 95% CI was (40.3%–49.5%).

Approximately 100 patients (≈15% of enrollment) were anticipated to be in cohort B, and ≈50 patients (≈8% of enrollment) were anticipated to be in cohort C.

## Results

Of the 1501 patients screened, 650 were enrolled (454 United States, 196 Europe). A study flow diagram and screen failure details are available in the Data Supplement. The completion rate through final follow-up was high (94.6%), and the attrition rate (5.4%) was better than the expected 10%. Of the 35 patients that did not complete the study, 5 withdrew consent, 2 were withdrawn by a study investigator, 25 were lost to follow-up, and 3 did not complete for other reasons.

Baseline data are presented in Table 1. Median age was 70 years (range, 22–96 years), and 54% were female. Most patients (52.8%) were transferred from another hospital, and 49.2% received intravenous recombinant tissue-type plasminogen activator before thrombectomy. Strokes were witnessed, wakeup, or unwitnessed in 52.2%, 10.0%, and 37.8% of cases, respectively. Median time from onset to door was 193 minutes (interquartile range, 83–378), and median time from door to puncture was 68 minutes (interquartile range, 40–102). Median ASPECTS was 8, and median posterior circulation ASPECTS was 9.

**Table 1.**
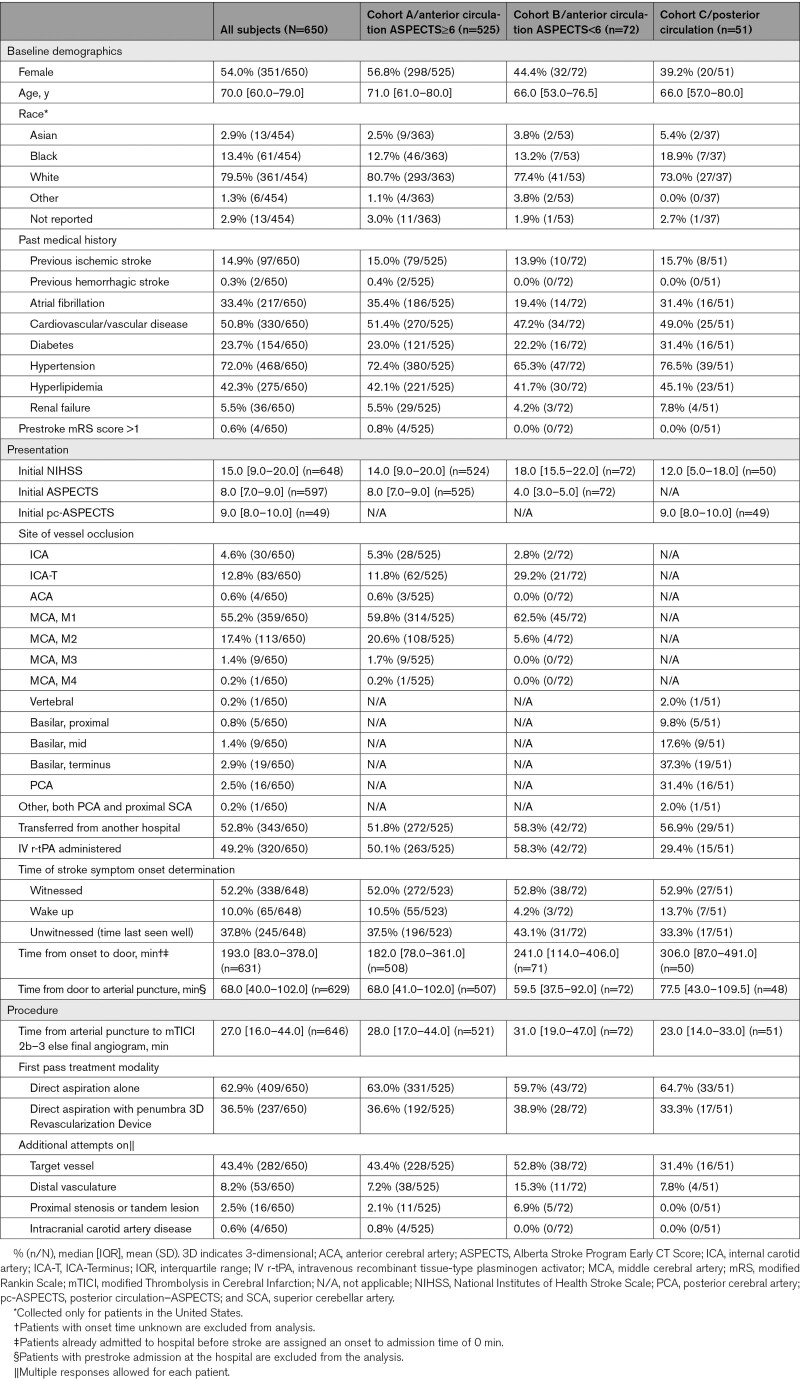
Baseline Characteristics and Procedural Data

Most patients had an anterior circulation LVO (92.2%) and the remaining 7.8% had a posterior circulation LVO. Two patients with anterior circulation LVO were missing ASPECTS and, therefore, not assigned to any cohort. The most common anterior LVO locations were the M1 middle cerebral artery (59.9%), M2 middle cerebral artery (18.9%), and internal carotid artery terminus (13.9%). The most common posterior LVO locations were the basilar terminus (37.3%), posterior cerebral artery (31.4%), and middle portion of the basilar artery (17.6%).

Procedural data are available in Table 1. Frontline treatment was aspiration alone in 62.9% of cases and aspiration combined with the 3D Revascularization Device in 36.5% of cases. For frontline aspiration alone cases, the most common reperfusion catheters chosen were the ACE68 (43.5%) and the JET7 (37.9%). For frontline combined technique cases, the most common reperfusion catheters chosen were the JET7 (49.4%) and the ACE68 (26.2%). In 8 cases, the 3D Revascularization Device was used with a non-Penumbra catheter as frontline treatment. Median time from puncture to mTICI 2b to 3 reperfusion was 26 minutes (interquartile range, 15–40). Additional device use details and preprocedural and procedural time metrics are available in the Data Supplement.

### Outcomes—All Patients

Outcome information is available in Table 2. Successful revascularization (mTICI 2b–3) was achieved in 56.8% (368/648) of cases postfirst pass, 76.4% (496/649) post-Penumbra System, and 87.8% (571/650 [95% CI, 85.3%–90.4%]) postprocedure. Functional outcome (mRS score 0–2) at 90 days was 55.8% (342/613 [95% CI, 51.9%–59.7%]). The Figure shows a full distribution of mRS scores at 90 days.

**Table 2.**
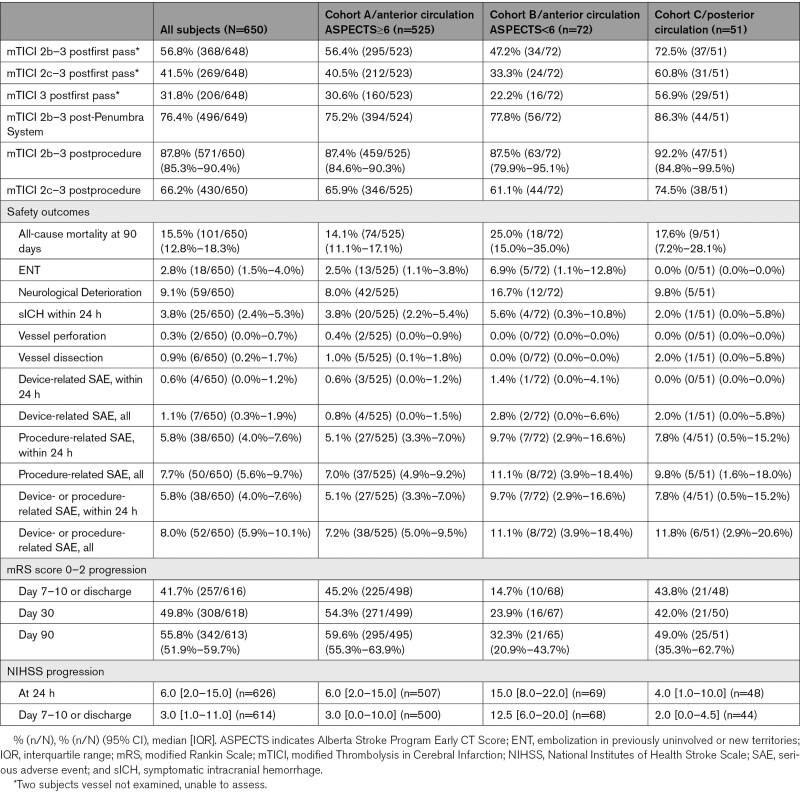
Outcomes

**Figure.**
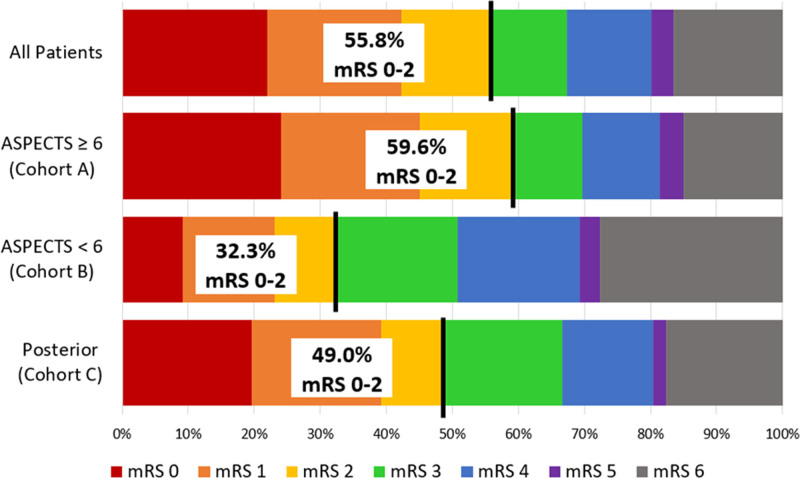
Distribution of the modified Rankin Scale (mRS) scores at 90 d for the full study population (all patients) and the pre-specified cohorts (Cohort A, ASPECTS [Alberta Stroke Program Early CT Score] 6-10; cohort B, ASPECTS 0-5, large core infarct; and cohort C, posterior circulation occlusion).

Vessel perforation occurred in 0.3% (2/650) of cases, vessel dissection in 0.9% (6/650), and ENT in 2.8% (18/650). Device-related SAE within 24 hours occurred in 0.6% (4/650) of cases, procedure-related SAE within 24 hours in 5.8% (38/650), neurological deterioration in 9.1% (59/650), and sICH within 24 hours in 3.8% (25/650). Overall, 36.5% (237/650) of patients experienced any ICH within 24 hours. Using ECASS classification (Table 3), rate of PH-2 was 3.1% (20/650), and rate of SAH was 8.2% (53/650). All-cause mortality at 90 days was 15.5% (101/650 [95% CI, 12.8%–18.3%]).

**Table 3.**
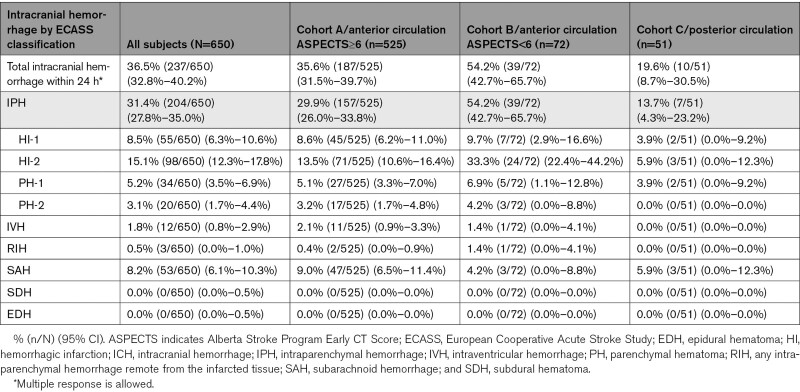
Intracranial Hemorrhage Within 24 Hours, ECASS Classification

### Outcomes—Cohort A (Anterior LVO With ASPECTS≥6)

For cohort A, mTICI 2b to 3 was achieved in 56.4% (295/523) of cases postfirst pass, 75.2% (394/524) post-Penumbra System, and 87.4% (459/525 [95% CI, 84.6%–90.3%]) postprocedure. At 90 days, mRS score 0 to 2 was 59.6% (295/495 [95% CI, 55.3%–63.9%]).

Vessel perforation occurred in 0.4% (2/525) of cases, vessel dissection in 1.0% (5/525), and ENT in 2.5% (13/525). Device-related SAE within 24 hours occurred in 0.6% (3/525) of cases, procedure-related SAE within 24 hours in 5.1% (27/525), neurological deterioration in 8.0% (42/525), and sICH within 24 hours in 3.8% (20/525). Overall, 35.6% (187/525) of cohort A experienced any ICH within 24 hours. Using ECASS classification (Table 3), rate of PH-2 was 3.2% (17/525), and rate of SAH was 9.0% (47/525). All-cause mortality at 90 days was 14.1% (74/525 [95% CI, 11.1%–17.1%]).

### Outcomes—Cohort B (Anterior LVO With ASPECTS<6)

For cohort B, mTICI 2b to 3 was achieved in 47.2% (34/72) of cases postfirst pass, 77.8% (56/72) post-Penumbra System, and 87.5% (63/72 [95% CI, 79.9%–95.1%]) postprocedure. At 90 days, mRS score 0 to 2 was 32.3% (21/65 [95% CI, 20.9%–43.7%]).

Vessel perforation occurred in 0.0% (0/72) of cases, vessel dissection in 0.0% (0/72), and ENT in 6.9% (5/72). Device-related SAE within 24 hours occurred in 1.4% (1/72) of cases, procedure-related SAE within 24 hours in 9.7% (7/72), neurological deterioration in 16.7% (12/72), and sICH within 24 hours in 5.6% (4/72). Overall, 54.2% (39/72) of cohort B experienced any ICH within 24 hours. Using ECASS classification (Table 3), rate of PH-2 was 4.2% (3/72), and rate of SAH was 4.2% (3/72). All-cause mortality at 90 days was 25.0% (18/72 [95% CI, 15.0%–35.0%]).

### Outcomes—Cohort C (Posterior LVO)

For cohort C, mTICI 2b to 3 was achieved in 72.5% (37/51) of cases postfirst pass, 86.3% (44/51) post-Penumbra System, and 92.2% (47/51 [95% CI, 84.8%–99.5%]) postprocedure. At 90 days, mRS score 0 to 2 was 49.0% (25/51 [95% CI, 35.3%–62.7%]).

Vessel perforation occurred in 0.0% (0/51) of cases, vessel dissection in 2.0% (1/51), and ENT in 0.0% (0/51). Device-related SAE within 24 hours occurred in 0.0% (0/51) of cases, procedure-related SAE within 24 hours in 7.8% (4/51), neurological deterioration in 9.8% (5/51), and sICH within 24 hours in 2.0% (1/51). Overall, 19.6% (10/51) of cohort C experienced any ICH within 24 hours. Using ECASS classification (Table 3), rate of PH-2 was 0.0% (0/51), and rate of SAH was 5.9% (3/51). All-cause mortality at 90 days was 17.6% (9/51 [95% CI, 7.2%–28.1%]).

## Discussion

To date, COMPLETE is the largest prospective study evaluating the real-world experience of using the Penumbra System as the frontline MT approach in patients with LVO-AIS. In the anterior circulation LVO with ASPECTS≥6 cohort, the study demonstrated a postprocedure revascularization success (mTICI 2b–3) rate of 87.4% and an excellent first pass effect (after a single attempt with the Penumbra System, mTICI 2b to 3, mTICI 2c to 3, and mTICI 3 was achieved in 56.4%, 40.5%, and 30.6% of patients, respectively). These angiographic results translated to a high proportion of patients with good clinical outcomes, with close to 60% of patients regaining functional independence at 90 days. Safety results demonstrated low rates of sICH (3.8%) and all-cause mortality (14.1%). The observed postprocedure mTICI 2b to 3 rate (87.4% [95% CI, 84.6 %–90.3%]) fell within the range of our predetermined expected rate (85% [95% CI, 81.9%–88.1%]). The 95% CI of 84.6% to 90.3% is consistent with the 85% to 90% target effect mentioned by Lin and Saver^18^ in their publication regarding minimal clinically important difference for substantial reperfusion. The observed 90-day mRS score 0 to 2 success rate (59.6% [95% CI, 55.3%–63.9%]) was better than expected (predetermined expected rate was 45% [95% CI, 40.3%–49.5%]).

### Real-World COMPLETE Registry Versus Randomized Clinical Trials

To accurately capture real-world performance, COMPLETE involved a large number of sites and a range of operator of experience levels. Prior randomized clinical trials, COMPASS and ASTER, selected high-volume sites with experienced operators.^13,16^ In COMPASS, the rates of mTICI 2b to 3 at end of procedure, functional independence (mRS score 0–2), sICH, and all-cause mortality were 92%, 52%, 6%, and 22%, respectively.^16^ In ASTER, the rates of mTICI 2b to 3 at end of procedure, functional independence (mRS score 0–2), sICH, and all-cause mortality were 85%, 45%, 5%, and 19%, respectively.^13^ As comparison, in the anterior circulation LVO with ASPECTS≥6 cohort of our COMPLETE real-world registry, the rates of mTICI 2b to 3 at end of procedure, functional independence (mRS score 0–2), sICH, and all-cause mortality were 87%, 60%, 4%, and 14%, respectively. Despite including a wide range of clinical site and operator experience levels, the COMPLETE registry’s angiographic, functional independence, and safety outcomes were comparable to, or better than prior randomized trials. Additionally, both COMPASS and ASTER restricted enrollment to patients presenting within 6 hours of symptom onset whereas COMPLETE did not restrict by time from symptom onset.^13,16^

Several factors contributed to the excellent angiographic, functional, and safety outcomes found in our COMPLETE registry. Technical advances over the years have made aspiration catheters more trackable and easier to navigate. The wide availability of larger inner diameter aspiration catheters (eg, JET 7) and newer generation pump (eg, ENGINE pump) improves the efficiency of aspiration and may have led to the high rate of successful revascularization seen in COMPLETE. Additionally, the functional independence rate (59.6%) is one of the highest reported in the literature for patients with anterior circulation LVO with ASPECTS≥6 treated frontline with Penumbra System. This can be partially explained by the high rate of first pass successful revascularization to mTICI 2b to 3 (56.4%) and partially explained by better patient selection. Since the series of positive MT trials,^3–8^ advanced imaging to screen patients is more prevalent,^6,19^ and many stroke teams have established clear patient selection criteria for MT. These improvements potentially contributed to the higher rate of good clinical outcome, lower rate of sICH, and lower rate of all-cause mortality found in this study.

### Additional Subgroup Outcomes in the Complete Real-World Registry

Despite the large sample size (N=650), the current study had few patients enrolled with anterior circulation LVO and ASPECTS<6 (n=72), or posterior circulation LVO (n=51). These cohorts yielded several unexpected observations. Functional independence at 90 days was better than expected (32.3% for low ASPECTS, and 49.0% for posterior LVO). These rates are numerically higher than those reported by Kaesmacher et al^20^ in a recent multicenter pooled analysis of 237 patients with low ASPECTS and those reported by Deb-Chatterji et al^21^ in a subgroup analysis of 152 patients from the German Stroke Registry–Endovascular Treatment with low ASPECTS. In the Kaesmacher et al^20^ and Deb-Chatterji et al^21^ studies, functional independence was achieved in 24.6% of patients and 21.7% of patients, respectively. Additionally, our study’s low ASPECTS cohort had favorable rates of sICH (5.6%) and all-cause mortality (25.0%) when compared with Kaesmacher et al^20^ (sICH 7.2%, all-cause mortality 40.1%) and Deb-Chatterji et al^21^ (sICH not reported, all-cause mortality 44.7%).

There are limited prospective independently adjudicated data involving exclusively Penumbra System as frontline therapy on posterior circulation LVOs. In our cohort, we had a better than expected rate of functional independence at 90 days (49.0%) and low rates of sICH (2.0%) and all-cause mortality at 90 days (17.6%). This compares favorably to a recent systematic review of posterior circulation LVO MT treatment which demonstrated 38% favorable outcomes at 90 days and 30% mortality.^22^

The high rate of successful revascularization achieved in COMPLETE’s posterior circulation cohort (92.2% mTICI 2b–3 postprocedure and first pass mTICI 2b–3, 2c–3, and 3 of 72.5%, 60.8%, and 56.9%, respectively, versus 56.4%, 40.5%, and 30.6%, respectively, in the anterior circulation LVO with ASPECTS≥6 cohort) is an important finding that warrants further investigation.

### Strengths and Limitations

Limitations of the COMPLETE registry include the lack of a randomized comparison group, the ability to enroll emergent cases after the procedure (potentially biasing toward better outcomes as surrogates of patients suffering severe strokes may be less willing to consent to participate), and the absence of an independent certified assessor to evaluate mRS outcomes. Additionally, not all consecutive patients were enrolled (eg, if informed consent was not obtained within the prespecified timeframe or if a patient declined to participate). Strengths of the COMPLETE registry include the prospective nature, the large sample size, the use of an imaging core lab and independent medical reviewers, and the inclusion of a variety of sites and operator experience levels to more accurately reflect real-world clinical practice.

### Conclusions

The COMPLETE registry found that use of the Penumbra System for frontline MT treatment of patients with LVO-AIS in a real-world setting was associated with angiographic, clinical, and safety outcomes that were comparable to prior randomized clinical trials that utilized stringent site and operator selection criteria.

## Article Information

### Acknowledgments

We acknowledge the contributions of all research coordinators and investigators that contributed to the COMPLETE (International Acute Ischemic Stroke Registry With the Penumbra System Aspiration Including the 3D Revascularization Device) Registry’s data collection and entry process. The authors additionally acknowledge Penumbra employees Hee Jung Lee, MS, and Nam Nguyen, MS, for statistical assistance, and Vincent Ho, MD, for writing assistance.

### Sources of Funding

This study was funded by Penumbra, Inc (Alameda). Drs Zaidat, Fifi, and A.E. Hassan were involved in study design. The sponsor was responsible for database setup, site monitoring, data management, and statistical analysis.

### Disclosures

Dr Zaidat reports grants and personal fees from Penumbra and four other companies; patents pending or issued for aneurysm and stroke device(s). Dr Fifi reports personal fees and grants from Penumbra, Stryker, and Microvention; personal fees from Cerenovus; ownership interest in Imperative Care. Dr Doerfler reports other from Penumbra (sponsored the study and made payments to the institution) during the conduct of the study. Dr Singh reports other from Penumbra (clinical trial fees) during the conduct of the study. Dr Katz reports grants from Penumbra, Siemens Healthineers, National Institutes of Health (NIH)/National Institute of Neurological Disorders and Stroke (NINDS), and Medtronic. Dr Sheth reports personal fees (core imaging lab) from Penumbra during the conduct of the study. Dr Yoo reports personal fees (core imaging lab) from Penumbra during the conduct of the study; grants and personal fees from Cerenovus; grants from Penumbra, Medtronic, and Stryker; personal fees from Vesalio; equity interest in Insera Therapeutics. Dr Hassan reports personal fees from Medtronic, Stryker, Microvention, GE Healthcare, Vizai, Scientia, Penumbra, Cerenovus, Rapid Medical, and Balt. The other authors report no conflicts.

### Supplemental Materials

Online Listing I

Online Figure I

Online Tables I–VII

## Supplementary Material


