# Supplementary material for: Endovascular Treatment of Acute Ischemic Stroke With the Penumbra System in Routine Practice: COMPLETE Registry Results
Source: Stroke. 2021 Sep 22;53(3):769–78. doi: 10.1161/STROKEAHA.121.034268 (PMC8884134; doi:10.1161/STROKEAHA.121.034268)
Supplement: Supplementary file 1 [file str-53-0769-s001.pdf]

Endovascular Treatment of Acute Ischemic Stroke with the Penumbra System in Routine Practice: COMPLETE Registry Results

SUPPLEMENTAL MATERIAL

Corresponding Author:

Osama O. Zaidat, MD, MS
Neuroscience & Stroke Director
Professor, Northeast Ohio Medical University (NeoMed)
Mercy Health St. Vincent Medical Center
2222 Cherry Street, Suite M200, Toledo OH 43608
Email: oozaidat@mercy.com

Supplemental Materials

Supplemental Listing I: Institutional Review Board/ Ethics Committees
Supplemental Figure I: Patient Flow Diagram
Supplemental Table I: RECORD Checklist
Supplemental Table II: Investigators of the COMPLETE Registry
Supplemental Table III: Device Use Details
Supplemental Table IV: Pre-procedure and Procedure Time Metrics
Supplemental Table V: Screen Failure Details
Supplemental Table VI: Sensitivity Analysis for mRS 0-2 at 90 days
Supplemental Table VII: Pooling Across Centers Adjusted Analysis (per Protocol Population)

Supplemental Listing I: Institutional Review Board/ Ethics Committees

The study was approved by the following Institutional Review Boards and Ethics Committees: Comité de protection des personnes Sud-Ouest et Outre-Mer 4, Friedrich-Alexander-Universität Erlangen-Nürnberg, Ethikkommission der Universität zu Lübeck, Ethikkommission bei der Sächsischen Landesärztekammer, Medizinische Fakultät/Universitätsklinikum Magdeburg A. ö. R., Komisja Bioetyczna przy Uniwersytecie Medycznym w Lublinie, Independet Ethics Committee at SBHI [State Budgetary Hospital] n.a. I. V. Davydovsky MHD [Moscow Health Department], Local Ethics Committee of SPb GBUZ ''City Multidisciplinary Hospital No. 2'', Local Ethics Committee at the City Clinical Hospital No. 1 named after N.I. Pirogov No. 9, Comité Ético de Investigación Clínica (CEIC) del Hospital Universitario Vall d'Hebron, CEIC del Hospital Virgen de la Arrixaca, Western Institutional Review Board, University of Miami Human Subject Research Office, Cedars-Sinai, Office of Research Compliance and Quality Improvement, UTHSC (University of Tennessee Health Science Center) IRB, KUMC (University of Kansas Medical Center) Human Research Protection Program IRB, HCA – HealthONE IRB, AdventHealth Orlando IRB, Prisma Health-Midlands IRB, Sutter Health IRB, St Joseph Health IRB, Human Research Committee Partners HealthCare Systems Inc., Dignity Health, NYU School of Medicine's Office of Science and Research Institutional Review Board, MetroWest Medical Center IRB, Ochsner Clinic Foundation IRB, Houston Methodist Research Institute, Naples Community Hospital IRB, SSMSTL (SSM Health Care St Louis) IRB, UT (University of Tennessee)-College of Medicine IRB, Covenant Health IRB, Mercy Health North LLC Adult IRB Research Oversight and Education

Supplemental Figure I: Patient Flow Diagram

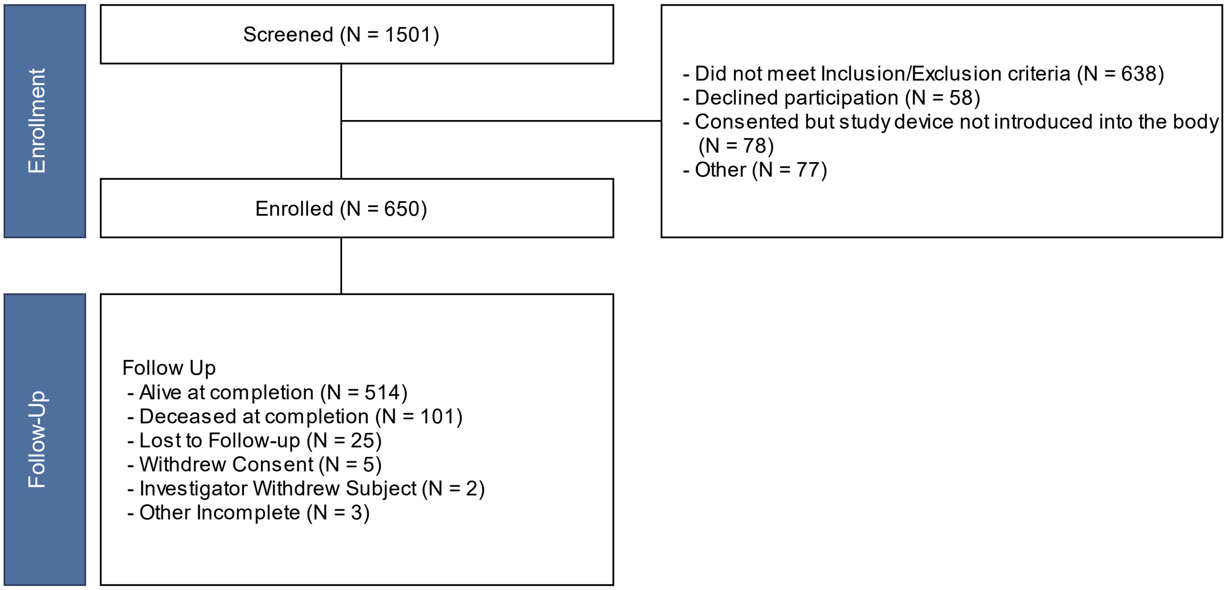

Supplemental Table I: RECORD Checklist

	Item No.	STROBE items	Location in manuscript where items are reported	RECORD items	Location in manuscript where items are reported
**Title and abstract**					
	1	(a) Indicate the study's design with a commonly used term in the title or the abstract (b) Provide in the abstract an informative and balanced summary of what was done and what was found	(a) Abstract, background & purpose section<br><br>(b) Abstract	RECORD 1.1: The type of data used should be specified in the title or abstract. When possible, the name of the databases used should be included.<br><br>RECORD 1.2: If applicable, the geographic region and timeframe within which the study took place should be reported in the title or abstract.<br><br>RECORD 1.3: If linkage between databases was conducted for the study, this should be clearly stated in the title or abstract.	(1.1) Abstract, methods & results sections<br><br>(1.2) Abstract, methods section<br><br>(1.3) N/A
**Introduction**					
Background rationale	2	Explain the scientific background and rationale for the investigation being reported	Introduction		
Objectives	3	State specific objectives, including any prespecified hypotheses	Introduction paragraph 4		
**Methods**					
Study Design	4	Present key elements of study design early in the paper	Methods paragraph 1		
Setting	5	Describe the setting, locations, and relevant dates, including periods of recruitment, exposure, follow-up, and data collection	Methods paragraph 1		
Participants	6	*(a) Cohort study* - Give the eligibility criteria, and the sources and methods of selection of participants. Describe methods of follow-up<br>*Case-control study* - Give the eligibility criteria, and the sources and methods of case ascertainment	(a) Methods paragraph 2, 3. Methods, endpoints section paragraph 3<br><br>(b) N/A	RECORD 6.1: The methods of study population selection (such as codes or algorithms used to identify subjects) should be listed in detail. If this is not possible, an explanation should be provided.	(6.1) Methods paragraph 4

		and control selection. Give the rationale for the choice of cases and controls<br>*Cross-sectional study* - Give the eligibility criteria, and the sources and methods of selection of participants<br><br>*(b) Cohort study* - For matched studies, give matching criteria and number of exposed and unexposed<br>*Case-control study* - For matched studies, give matching criteria and the number of controls per case		RECORD 6.2: Any validation studies of the codes or algorithms used to select the population should be referenced. If validation was conducted for this study and not published elsewhere, detailed methods and results should be provided.<br><br>RECORD 6.3: If the study involved linkage of databases, consider use of a flow diagram or other graphical display to demonstrate the data linkage process, including the number of individuals with linked data at each stage.	(6.2) N/A<br><br>(6.3) N/A
Variables	7	Clearly define all outcomes, exposures, predictors, potential confounders, and effect modifiers. Give diagnostic criteria, if applicable.	Methods, endpoints section	RECORD 7.1: A complete list of codes and algorithms used to classify exposures, outcomes, confounders, and effect modifiers should be provided. If these cannot be reported, an explanation should be provided.	Methods, endpoints section, statistical analysis section
Data sources/ measurement	8	For each variable of interest, give sources of data and details of methods of assessment (measurement).<br>Describe comparability of assessment methods if there is more than one group	Methods paragraph 1, 6. Methods, endpoints section. Methods, study committees section.		
Bias	9	Describe any efforts to address potential sources of bias	Methods, paragraph 4 and study committees section.		
Study size	10	Explain how the study size was arrived at	Methods, statistical analysis section		
Quantitative variables	11	Explain how quantitative variables were handled in the analyses. If applicable, describe which groupings were chosen, and why	Methods, paragraph 6 and statistical analysis section		
Statistical methods	12	(a) Describe all statistical methods, including those used to control for confounding	(a) Methods, statistical analysis section		

		(b) Describe any methods used to examine subgroups and interactions<br>(c) Explain how missing data were addressed<br>(d) *Cohort study* - If applicable, explain how loss to follow-up was addressed<br>*Case-control study* - If applicable, explain how matching of cases and controls was addressed<br>*Cross-sectional study* - If applicable, describe analytical methods taking account of sampling strategy<br>(e) Describe any sensitivity analyses	(b) Methods, paragraph 6<br><br>(c) Methods, statistical analysis section & online supplemental.<br><br>(d) Methods, statistical analysis section & online supplemental.<br><br>(e) Methods, statistical analysis section & online supplemental.		
Data access and cleaning methods		..		RECORD 12.1: Authors should describe the extent to which the investigators had access to the database population used to create the study population.<br><br>RECORD 12.2: Authors should provide information on the data cleaning methods used in the study.	(12.1) Methods, paragraph 4<br><br>(12.2) Methods, paragraph 4
Linkage		..		RECORD 12.3: State whether the study included person-level, institutional-level, or other data linkage across two or more databases. The methods of linkage and methods of linkage quality evaluation should be provided.	(12.3) N/A
**Results**					
Participants	13	(a) Report the numbers of individuals at each stage of the study (*e.g.*, numbers potentially eligible, examined for eligibility, confirmed eligible, included in the study, completing follow-up, and analysed)<br>(b) Give reasons for non-participation at each stage.	(a) Results, paragraph 1<br><br>(b) Results, paragraph 1 and supplemental table V	RECORD 13.1: Describe in detail the selection of the persons included in the study (*i.e.,* study population selection) including filtering based on data quality, data availability and linkage. The selection of included persons can be described in the text and/or by means of the study flow diagram.	(13.1) Results, paragraph 1, supplemental table V, and supplemental figure I

		(c) Consider use of a flow diagram	(c) Supplemental figure I		
Descriptive data	14	(a) Give characteristics of study participants (*e.g.*, demographic, clinical, social) and information on exposures and potential confounders<br>(b) Indicate the number of participants with missing data for each variable of interest<br>(c) *Cohort study* - summarise follow-up time (*e.g.*, average and total amount)	(a) Results, paragraph 1 and 2. Table 1<br><br>(b) Table 1<br><br>(c) Results, outcomes section. Table 2.		
Outcome data	15	*Cohort study* - Report numbers of outcome events or summary measures over time<br>*Case-control study* - Report numbers in each exposure category, or summary measures of exposure<br>*Cross-sectional study* - Report numbers of outcome events or summary measures	Results, outcomes section. Table 2. Figure 1.		
Main results	16	(a) Give unadjusted estimates and, if applicable, confounder-adjusted estimates and their precision (e.g., 95% confidence interval). Make clear which confounders were adjusted for and why they were included<br>(b) Report category boundaries when continuous variables were categorized<br>(c) If relevant, consider translating estimates of relative risk into absolute risk for a meaningful time period	(a) Results, outcomes section. Table 2, table 3, figure 1.<br><br>(b) N/A<br><br>(c) N/A		
Other analyses	17	Report other analyses done—e.g., analyses of subgroups and interactions, and sensitivity analyses	Online supplemental for sensitivity analyses results		
**Discussion**					
Key results	18	Summarise key results with reference to study objectives	Discussion paragraph 1		

Limitations	19	Discuss limitations of the study, taking into account sources of potential bias or imprecision. Discuss both direction and magnitude of any potential bias	Discussion, strengths & limitations section	RECORD 19.1: Discuss the implications of using data that were not created or collected to answer the specific research question(s). Include discussion of misclassification bias, unmeasured confounding, missing data, and changing eligibility over time, as they pertain to the study being reported.	Discussion, strengths & limitations sections.
Interpretation	20	Give a cautious overall interpretation of results considering objectives, limitations, multiplicity of analyses, results from similar studies, and other relevant evidence	Summary/ conclusions section.		
Generalisability	21	Discuss the generalisability (external validity) of the study results	Discussion sections "real-world COMPLETE registry versus Randomized Clinical Trials", "Additional Subgroup Outcomes in the COMPLETE real-world registry", and "strengths and limitations"		
**Other Information**					
Funding	22	Give the source of funding and the role of the funders for the present study and, if applicable, for the original study on which the present article is based	Sources of Funding section.		
Accessibility of protocol, raw data, and programming code		..		RECORD 22.1: Authors should provide information on how to access any supplemental information such as the study protocol, raw data, or programming code.	Methods, paragraph 1

Supplemental Table II: Investigators of the COMPLETE Registry

Sites Located in EU	
**Institution (Name & Address)**	**Investigator(s)**
Foundation Ophthalmic Adolphe De Rothschild – 29 Rue Manin – 75019 – Paris – France	Michel Piotin (PI)<br>Raphaël Blanc (Sub-I)<br>Jean-Philippe Desilles (Sub-I)<br>Gabriele Ciccio (Sub-I)<br>Stanislas Smajda (Sub-I)
CHU de Bordeaux – Hôpital Pellegrin – Place Amélie Raba Léon – 33000 – Bordeaux – France	Jérôme Berge (PI)<br>Gaultier Marnat (Sub-I)<br>Xavier Barreau (Sub-I)<br>Patrice Ménégon (Sub-I)<br>Florent Gariel (Sub-I)
Universitätsklinikum Erlangen – Schwabachanlage 6 – 91054 – Erlangen – Germany	Arnd Dörfler (PI)<br>Tobias Engelhorn (Sub-I)<br>Philipp Gölitz (Sub-I)<br>Manuel Schmidt (Sub-I)<br>Stefan Lang (Sub-I)<br>Iris Mühlen (Sub-I)<br>Julie Rösch (Sub-I)<br>Michael Knott (Sub-I)<br>Hannes Lücking (Sub-I)<br>Philip Hoelter (Sub-I)
Universitätsklinikum Schleswig-Holstein Campus Lübeck – Ratzeburger Allee 160 – 23538 – Lübeck – Germany	Peter Schramm (PI)<br>Alexander Neumann (Sub-I)<br>Thomas Eckey (Sub-I)<br>Tobias Boppel (Sub-I)
Klinikum Chemnitz gGmbH – Flemmingstraße 2 – 09116 – Chemnitz – Germany	Jörg Thalwitzer (PI)<br>Mike Krah (Sub-I)<br>Jens Schwarze (Sub-I)<br>Bettina Manikowski (Sub-I)<br>Philipp Ladig (Sub-I)<br>Nathanael Albert (Sub-I)<br>Carmen Otto (Sub-I)<br>Anne Lemme (Sub-I)<br>Silvio Brandt (Sub-I)
Charité – Universitätsmedizin Berlin – Charitéplatz 1 – 10117 – Berlin – Germany	Georg Bohner (PI)<br>Justus Kleine (Sub-I)<br>Eberhard Siebert (Sub-I)<br>Hans-Christian Bauknecht (Sub-I)<br>Edzard Wiener (Sub-I)
Universitätsklinikum Magdeburg A. ö. R. – Leipziger Str. 44 – 39120 – Magdeburg – Germany	Oliver Beuing (PI)<br>Aneta Donitza (Sub-I)
Samodzielny Publiczny Szpital Kliniczny nr 4 w Lublinie – ul. Jaczewskiego 8 – 20 – 954 – Lublin – Poland	Tomasz Jargiełło (PI)<br>Michał Górnik (Sub-I)<br>Krzysztof Pyra (Sub-I)<br>Tomasz Roman (Sub-I)<br>Maciej Szajner (Sub-I)<br>Remigiusz Ficek (Sub-I)<br>Katarzyna Prus(Sub-I)<br>Sylwia Stachowicz (Sub-I)<br>Joanna Wojczal (Sub-I)<br>Michał Sojka (Sub-I)<br>Kinga Buraczyńska (Sub-I)<br>Piotr Luchowski (Sub-I)
Davidovsky Moscow City Hospital №23 – Yauzskaya Ulitsa, 11 – 109240 – Moscow – Russia	Dmitry Skrypnik (PI)<br>Kirill Anisimov (Sub-I)<br>Ekaterina Sukhanova (Sub-I)

Multidisciplinary City Hospital №2 – Uchebnyy Pereulok, 5 – 194354 – St. Petersburg – Russia	Eugene Shloydo (PI)<br>Kirill Kravchenko (Sub-I)<br>Konstantin Golikov (Sub-I)<br>Aleksey Sergeev (Sub-I)
City Clinical Hospital No. 1 named after N. I. Pirogov – Leninsky Ave, 8 - 119049 – Moscow – Russia	Anna Udovichenko (PI)<br>Roman Georgiev (Sub-I)<br>Eduard Gasparyan (Sub-I)<br>Svyatoslav Malov (Sub-I)<br>Eugeny Ploschenkov (Sub-I)
Hospital Universitari Vall d'Hebron – Passeig de la Vall d'Hebron 119-129 – 08035 – Barcelona – Spain	Alejandro Tomasello Weitz (PI)<br>Marc Ribó (Sub-I)<br>David Hernández (Sub-I)<br>David Rodriguez-Luna (Sub-I)<br>Álvaro García-Tornel (Sub-I)<br>Marta Rubiera (Sub-I)<br>Jesús Juega (Sub-I)<br>Marian Muchada (Sub-I)<br>Noelia Rodríguez Villatoro (Sub-I)<br>Sandra Boned Riera (Sub-I)<br>Jorge Pagola (Sub-I)<br>Matías Deck Román (Sub-I)<br>Carlos Piñana Plaza (Sub-I)
Hospital Clínico Universitario Virgen de la Arrixaca – Ctra. Madrid-Cartagena, s/n – 30120 – Murcia (El Palmar) – Spain	Joaquín Zamarro Parra (PI)<br>Guillermo Parrilla Reverter (Sub-I)<br>Mariano Espinosa de Rueda Ruiz (Sub-I)<br>Blanca García-Villalba Navaridas (Sub-I)<br>José Díaz Pérez (Sub-I)
**Sites located in US**	
**Institution (Name & Address)**	**Investigator(s)**
Advent Health Orlando<br>601 E. Rollins Street<br>Orlando, FL 32803	Frank Hellinger (PI)<br>Ravi Gandhi (Sub-I)<br>Michael Bellew (Sub-I)
Radiology Imaging Associates, P.C.<br>501 E. Hampden Avenue<br>Englewood, CO 80113	Benjamin Atchie (PI)<br>Richard Bellon (Sub-I)<br>Do Frei (Sub-I)<br>Ian Kaminsky (Sub-I)<br>Eric Arias (Sub-I)
Northwell Health<br>300 Community Drive<br>Manhasset, NY 11030	Jeffrey Katz (PI)<br>Henry Woo (Sub-I)<br>Ina Teron Molina (Sub-I)<br>Anand Patel (Sub-I)<br>Richard Libman (Sub-I)<br>Rohan Arora (Sub-I)
Fort Sanders Regional Medical Center<br>1901 West Clinch Avenue<br>Knoxville, TN 37916	Keith Woodward (PI)<br>Harry Hixson (Sub-I)
Palmetto Health Richland Hospital<br>5 Richland Medical Park Drive<br>Columbia, SC 29203	Cole Graham (PI)<br>Roham Moftakhar (Sub-I)
Houston Methodist Hospital<br>6565 Fannin Street<br>Houston, TX 77030	Orlando Diaz (PI)<br>Richard Klucznik (Sub-I)
Cedar Sinai Medical Center<br>8700 Beverly Boulevard<br>Los Angeles, CA 90048	Michael Alexander (PI)<br>Robert Jackson (Sub-I)<br>Matthew Padrick (Sub-I)<br>Oana Dumitrascu (Sub-I)<br>Konrad Schlick (Sub-I)<br>Edgar Olivas (Sub-I)<br>Shlee Song (Sub-I)<br>Zachary Barnard (Sub-I)

	Penelope Kornbluth (Sub-I)
University of Kansas Medical Center<br>Research Institute<br>3901 Rainbow Boulevard<br>Kansas City, KS 66160	Ernest John Madarang (PI)<br>Alan Reeves (Sub-I)
Mount Sinai Medical Center<br>1450 Madison Avenue<br>Klingenstein Clinical Center (KCC)<br>1 North<br>New York, NY 10029	Inder Paul Singh (PI)<br>Johanna Fifi (Sub-I)<br>Hazem Shoriah (Sub-I)<br>Kurt Yaeger (Sub-I)<br>Shahram Majidi (Sub-I)<br>Reade DeLacey (Sub-I)<br>Christopher Keller (Sub-I)<br>Tomoyoshi Shigematsu (Sub-I)<br>Gal Yaniv (Sub-I)<br>Thomas Oxley (Sub-I)
McAllen Medical Center<br>301 West Expressway 83<br>McAllen, TX 78503	Alexandros Georgiadis (PI)<br>Wondwossen Tekle (Sub-I)<br>Ameer Hassan (Sub-I)
Yale New Haven Hospital<br>2 Church Street South, Suite 401<br>New Haven, CT 06519	Charles Matouk (PI)<br>Ryan Herber (Sub-I)<br>Farhad Bahrassa (Sub-I)<br>Michele Johnson (Sub-I)
Erlanger Medical Center<br>975 East 3rd Street<br>Box 376<br>Chattanooga, TN 37403	Harris Hawk (PI)<br>Steven Quarfordt (Sub-I)<br>Michael Nichols (Sub-I)<br>Justin Calvert (Sub-I)
Jackson Memorial Hospital<br>1611 NW 12th Avenue<br>Miami, FL 33136	Robert Starke (PI)<br>Dileep Yavagal (Sub-I)<br>Eric Peterson (Sub-I)
The Valley Hospital<br>223 N. Van Dien Avenue<br>Ridgewood, NJ 07450	Dorothea Altschul (PI)<br>Ahsan Sattar (Sub-I)
Brigham & Women's Hospital<br>75 Francis Street<br>Boston, MA 02115	Mohammed Ali Aziz-Sultan (PI)<br>Daniel Large (Sub-I)<br>Nirav Patel (Sub-I)<br>Karen Chen (Sub-I)<br>Habibullah Ziayee (Sub-I)<br>Rose Du (Sub-I)<br>Kai Frerichs (Sub-I)
Swedish Medical Center-Cherry Hill<br>550-17th Avenue<br>Seattle, WA 98122	Akshal Patel (PI)<br>Stephen Monteith (Sub-I)<br>Yince Loh (Sub-I)<br>Mohammed Aref (Sub-I)
Thomas Jefferson University Hospital<br>125 S. 9th Street<br>Sheridan Building<br>Philadelphia, PA 19107	Stavropoula Tjoumakaris (PI)<br>Nabeel Herial (Sub-I)<br>Pacal Jabbour (Sub-I)<br>Robert Rosenwasser (Sub-I)<br>Michael Reid Gooch (Sub-I)<br>Hekmat Zarzour (Sub-I)
Los Robles Hospital<br>215 W Janss Road<br>Thousand Oaks, CA 91360	Muhammad Taqi (PI)
Semmes Murphey<br>Methodist University Hospital<br>1265 Union Avenue<br>Memphis, TN 38104	Daniel Hoit (PI)<br>Adam Arthur (Sub-I)<br>Lucas Elijovich (Sub-I)<br>Christopher Nickele (Sub-I)<br>Violiza Inoa (Sub-I)<br>Nitin Goyal (Sub-I)<br>Radmehr Torabi (Sub-I)<br>David Dornbos (Sub-I)<br>Jeremy Peterson (Sub-I)

Mercy San Juan Medical Center<br>6501 Coyle Avenue<br>Carmichael, CA 95608	Lucian Maidan (PI)<br>George Luh (Sub-I)
SSM St. Clare Healthcare<br>1015 Bowles Avenue<br>Fenton, MO 63026	Ashish Nanda (PI)<br>Sushant Kale (Sub-I)<br>Amer Alshekhlee (Sub-I)
St Jude Medical Center<br>101 E. Valencia Mesa Drive<br>Fullerton, CA 92835	Hamed Farid (P)<br>Aaron Bress (Sub-I)<br>Cagin Senturk (Sub-I)
Ochsner Medical Center<br>1514 Jefferson Highway<br>New Orleans, LA 70121	Jim Milburn (PI)<br>Gabriel Vidal (Sub-I)<br>Paul Gulotta (Sub-I)<br>Edison Valle-Giler (Sub-I)
NYU Langone Hospital - Brooklyn<br>550 First Avenue New York, N.Y.<br>10016	David Turkel-Parrella (PI)<br>David S. Gordon (Sub-I)<br>Jeremy Liff (Sub-I)<br>Karthik Arcot (Sub-I)<br>Jeffrey Farkas (Sub-I)
Eden Medical Center<br>20103 Lake Chabot Road<br>Castro Valley, CA 94546	David Bonovich (PI)<br>Peter Adamczyk (Sub-I)
Valley Baptist Health System-<br>Harlingen<br>2101 Pease Street<br>Harlingen, TX 78550	Wondwossen Tekle (PI)<br>Ameer Hassan (Sub-I)
Mercy Health St. Vincent Medical<br>Center LLC.<br>2213 Cherry Street<br>Toledo, OH 43608	Osama Zaidat (PI)<br>Eugene Lin (Sub-I)<br>Mohammad Ezzeldin (Sub-I)<br>Ali Sultan-Qurraie (Sub-I)<br>Badar Alenzi (Sub-I)
Banner Desert Medical Center<br>1400 South Dobson Avenue Mesa,<br>Arizona 85202	Mohamed Teleb (PI)
Naples Community Hospital<br>350 7th Street N.<br>Naples, FL 34102	Mazen Abuawad (PI)<br>Ruta Viktoria Totoraitis (Sub-I)<br>Paul Richard (Sub-I)

Supplemental Table III: Device Use Details

	All Subjects (N=650)	Cohort A/ Anterior Circulation ASPECTS ≥6 (N=525)	Cohort B/ Anterior Circulation ASPECTS <6 (N=72)	Cohort C/ Posterior Circulation (N=51)
**Aspiration Source Used***				
Penumbra Pump MAX	35.2% (229/650)	35.4% (186/525)	31.9% (23/72)	37.3% (19/51)
Penumbra ENGINE	63.5% (413/650)	63.2% (332/525)	68.1% (49/72)	60.8% (31/51)
Other	2.2% (14/650)	2.5% (13/525)	0.0% (0/72)	2.0% (1/51)
**Neuron MAX 088 Long Sheath Use**	76.0% (494/650)	77.0% (404/525)	72.2% (52/72)	70.6% (36/51)
**Balloon Guide Catheter Use**	5.5% (36/650)	5.5% (29/525)	6.9% (5/72)	3.9% (2/51)
**Frontline treatment**				
**Direct Aspiration**	62.9% (409/650)	63.0% (331/525)	59.7% (43/72)	64.7% (33/51)
JET 7	23.8% (155/650)	23.2% (122/525)	29.2% (21/72)	23.5% (12/51)
JET D	4.0% (26/650)	4.4% (23/525)	2.8% (2/72)	2.0% (1/51)
ACE 68	27.4% (178/650)	27.2% (143/525)	26.4% (19/72)	27.5% (14/51)
ACE 64	1.2% (8/650)	1.5% (8/525)	0.0% (0/72)	0.0% (0/51)
ACE 60	1.1% (7/650)	1.1% (6/525)	1.4% (1/72)	0.0% (0/51)
4MAX	0.6% (4/650)	0.4% (2/525)	0.0% (0/72)	3.9% (2/51)
3MAX	4.8% (31/650)	5.1% (27/525)	0.0% (0/72)	7.8% (4/51)
**Penumbra Catheter with 3D Revascularization Device**	35.2% (229/650)	35.4% (186/525)	38.9% (28/72)	29.4% (15/51)
JET 7	18.0% (117/650)	17.9% (94/525)	20.8% (15/72)	15.7% (8/51)
JET D	4.0% (26/650)	4.0% (21/525)	4.2% (3/72)	3.9% (2/51)
ACE 68	9.5% (62/650)	9.5% (50/525)	11.1% (8/72)	7.8% (4/51)
ACE 64	0.9% (6/650)	1.0% (5/525)	0.0% (0/72)	2.0% (1/51)
ACE 60	0.9% (6/650)	1.1% (6/525)	0.0% (0/72)	0.0% (0/51)
4MAX	0.2% (1/650)	0.2% (1/525)	0.0% (0/72)	0.0% (0/51)
3MAX	1.7% (11/650)	1.7% (9/525)	2.8% (2/72)	0.0% (0/51)
**Other**	1.8% (12/650)	1.5% (8/525)	1.4% (1/72)	5.9% (3/51)

% (n/N)
*multiple responses allowed for each patient

Supplemental Table IV: Pre-Procedure and Procedure Time Metrics

Procedure Process Times	All Subjects (N=650)	Cohort A/ Anterior Circulation ASPECTS ≥6 (N=525)	Cohort B/ Anterior Circulation ASPECTS <6 (N=72)	Cohort C/ Posterior Circulation (N=51)
**Time from Onset to Door (mins)*†**	193.0 [83.0, 378.0] (n = 631)	182.0 [78.0, 361.0] (n = 508)	241.0 [114.0, 406.0] (n = 71)	306.0 [87.0, 491.0] (n = 50)
**Time from Door to Arterial Puncture (mins)‡**	68.0 [40.0, 102.0] (n = 629)	68.0 [41.0, 102.0] (n = 507)	59.5 [37.5, 92.0] (n = 72)	77.5 [43.0, 109.5] (n = 48)
**Time from Onset to Arterial Puncture (mins)***	276.0 [177.0, 485.0] (n = 631)	255.0 [174.0, 450.5] (n = 508)	332.0 [208.0, 484.0] (n = 71)	429.0 [202.0, 635.0] (n = 50)
**Time from Onset to mTICI 2b-3 first reached (mins)*§**	308.0 [205.0, 509.0] (n = 549)	290.0 [198.0, 474.0] (n = 439)	363.0 [240.0, 513.0] (n = 62)	454.0 [243.0, 610.0] (n = 46)
**Time from Arterial Puncture to mTICI 2b-3 first reached (mins)§**	26.0 [15.0, 40.0] (n = 567)	25.0 [15.0, 40.0] (n = 455)	29.0 [18.0, 44.0] (n = 63)	23.0 [14.0, 33.0] (n = 47)
**Time from Onset to mTICI 2b-3 else Final Angiogram (mins)***	313.0 [210.0, 525.0] (n = 627)	303.5 [203.0, 501.0] (n = 504)	358.0 [248.0, 513.0] (n = 71)	454.0 [243.0, 650.0] (n = 50)
**Time from Arterial Puncture to mTICI 2b-3 else Final Angiogram (mins)**	27.0 [16.0, 44.0] (n = 646)	28.0 [17.0, 44.0] (n = 521)	31.0 [19.0, 47.0] (n = 72)	23.0 [14.0, 33.0] (n = 51)
**Time from Onset to Final Angiogram (mins)***	321.0 [217.0, 543.0] (n = 626)	306.0 [210.5, 519.5] (n = 504)	363.0 [249.0, 532.0] (n = 70)	476.0 [278.0, 657.0] (n = 50)
**Time from Arterial Puncture to Final Angiogram (mins)**	33.0 [19.0, 56.0] (n = 645)	33.0 [19.0, 56.0] (n = 521)	39.0 [27.0, 57.0] (n = 71)	27.0 [15.0, 50.0] (n = 51)

Median [IQR]
mTICI, modified treatment in cerebral ischemia
*Patients with onset time unknown are excluded from analysis.
†Patients already admitted to hospital prior to stroke are assigned an onset to door time of 0 minute.
‡Patients with pre-stroke admission at the hospital are excluded from the analysis.
§Patients with final mTICI 2b-3 are included in analysis

Supplemental Table V: Screen Failure Details

Reason for Screen Failure	All Screen Failures (N=851)
**Inclusion criteria not met/ Exclusion criteria met***	75.0% (638/851)
• INC 1: Patient Age ≥ 18	• 4.5% (29/638)
• INC 2: Pre-stroke mRS 0-1	• 28.7% (183/638)
• INC 3: Patient experiencing acute ischemic stroke secondary to intracranial large vessel occlusion who are eligible for mechanical thrombectomy using the Penumbra System	• 6.7% (43/638)
• INC 4: Planned frontline treatment with Penumbra System	• 38.2% (244/638)
• INC 5: Signed informed consent per Institution Review Board/Ethics Committee	• 13.3% (85/638)
• EXC 1: Any comorbid disease or condition expected to compromise survival or ability to complete follow-up assessment through 90 days	• 8.0% (51/638)
• EXC 2: Currently participating in an investigational clinical trial that will confound registry endpoints	• 3.1% (20/638)
**Declined Participation**	6.8% (58/851)
**Consented but study device not introduced into the body**	9.2% (78/851)
**Other**	9.0% (77/851)

*Sixteen patients in this category had multiple inclusion criteria not met/ exclusion criteria met for screen failure

Supplemental Table VI: Sensitivity Analysis for mRS 0-2 at 90 days

Primary Endpoint(s) %(n/N) (95% CI)	All Subjects (N=650)	Cohort A (N=525)	Cohort B (N=72)	Cohort C (N=51)
Complete Day 90 Modified Rankin Scale 0-2	55.8% (342/613) (51.9%, 59.7%)	59.6% (295/495) (55.3%, 63.9%)	32.3% (21/65) (20.9%, 43.7%)	49.0% (25/51) (35.3%, 62.7%)
Complete Day 90 Modified Rankin Scale 0-2 (using multiple imputation method)*	55.8% (363/650) (52.0%, 59.7%)	59.8% (314/525) (55.6%, 64.0%)	31.9% (23/72) (21.2%, 42.7%)	49.0% (25/51) (35.3%, 62.7%)
Day 90 Modified Rankin Scale 0-2 by assigning group mRS 0-2	58.3% (379/650) (54.5%, 62.1%)	61.9% (325/525) (57.8%, 66.1%)	38.9% (28/72) (27.6%, 50.1%)	49.0% (25/51) (35.3%, 62.7%)
Day 90 Modified Rankin Scale 0-2 by assigning group mRS 3-6	52.6% (342/650) (48.8%, 56.5%)	56.2% (295/525) (51.9%, 60.4%)	29.2% (21/72) (18.7%, 39.7%)	49.0% (25/51) (35.3%, 62.7%)

*Based on multiple imputation using the fully conditional method with regression and 10 imputations, adjusted for age, IV tPA administration prior to procedure, baseline NIHSS, mTICI 2b-3 at final angiogram, target vessel location and mRS of 0 at baseline

Supplemental Table VII: Pooling Across Centers Adjusted Analysis (per Protocol Population)

Site, Adjusted odds ratio (95% CI)	Day 90 Modified Rankin Scale 0-2	Angiographic revascularization, mTICI 2b-3 per Core Lab else PI
00006	0.90 (0.49, 1.63)	1.57 (0.60, 4.11)
00038	0.72 (0.30, 1.73)	0.94 (0.28, 3.11)
00041	1.01 (0.55, 1.87)	1.10 (0.46, 2.65)
00108	1.35 (0.62, 2.97)	1.47 (0.43, 5.07)
00111	0.87 (0.42, 1.79)	0.64 (0.21, 1.94)
00122	1.45 (0.77, 2.74)	1.16 (0.46, 2.91)
00201	1.08 (0.58, 2.02)	1.32 (0.49, 3.52)
00225	0.79 (0.42, 1.50)	0.56 (0.23, 1.40)
00245	1.20 (0.56, 2.57)	1.37 (0.39, 4.80)
00274	0.85 (0.39, 1.84)	0.81 (0.26, 2.46)
00331	1.41 (0.60, 3.31)	1.35 (0.38, 4.75)
00332	0.83 (0.38, 1.82)	1.02 (0.32, 3.25)
00382	1.11 (0.52, 2.35)	1.56 (0.45, 5.38)
00387	0.91 (0.42, 1.96)	0.64 (0.19, 2.23)
00511	0.84 (0.49, 1.43)	0.45 (0.21, 0.97)
00555	1.27 (0.57, 2.84)	0.38 (0.11, 1.28)
00591	1.28 (0.62, 2.64)	2.00 (0.60, 6.62)
00769	0.61 (0.30, 1.25)	0.39 (0.15, 0.97)
00786	1.06 (0.55, 2.07)	0.95 (0.33, 2.72)
10004	1.54 (0.71, 3.32)	1.74 (0.52, 5.81)
10064	0.57 (0.23, 1.43)	1.06 (0.34, 3.33)
10167	0.79 (0.41, 1.51)	1.23 (0.45, 3.32)
70001	0.81 (0.43, 1.53)	0.72 (0.28, 1.83)
70007	1.13 (0.54, 2.38)	1.50 (0.43, 5.19)
Other Group 1	0.92 (0.48, 1.73)	0.41 (0.15, 1.10)
Other Group 2	1.55 (0.74, 3.27)	1.20 (0.45, 3.25)
Other Group 3	1.03 (0.52, 2.05)	1.14 (0.37, 3.51)

Note: Sites with less than 10 subjects are combined. Other Group 1-3 are combined sites in sequential order until pooled n is less than or equal to 30.
Endpoint adjusted odds ratios using generalized hierarchical linear mixed model adjusting for Age, NIHSS, IV tPA, Target Vessel Location, Baseline mRS of 0, ASPECTS less than 6 and onset to admission time with site as random effect.
