# Supplementary figures and images for: Endovascular Treatment of Acute Ischemic Stroke With the Penumbra System in Routine Practice: COMPLETE Registry Results
Source: Stroke. 2021 Sep 22;53(3):769–78. doi: 10.1161/STROKEAHA.121.034268 (PMC8884134; doi:10.1161/STROKEAHA.121.034268)

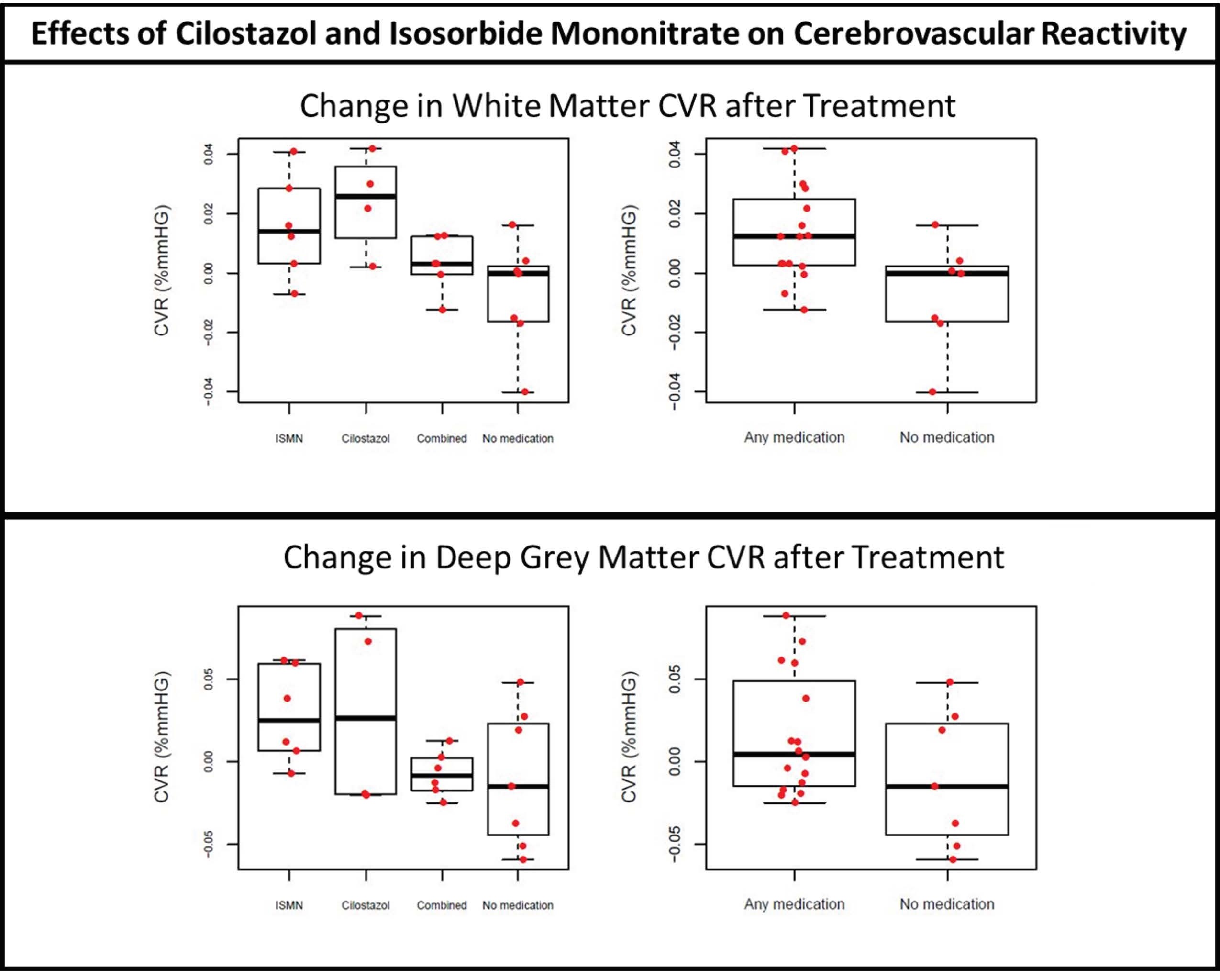

Supplement: Supplementary file 2 [file str-53-0769-s002.jpg]
