# Supplementary material for: Endovascular Treatment of Acute Ischemic Stroke With the Penumbra System in Routine Practice: COMPLETE Registry Results
Source: Stroke. 2021 Sep 22;53(3):769–78. doi: 10.1161/STROKEAHA.121.034268 (PMC8884134; doi:10.1161/STROKEAHA.121.034268)
Supplement: Supplementary file 3 [file str-53-0769-s003.pdf]

DocuSign Envelope ID: 05B57D1F-49A7-4F4D-BBA6-746ABE31BFD5

American Stroke Association.
A division of the American Heart Association.

Acknowledgment Permission Form

Journal Stroke
Manuscript Number STROKE/2021/034268R2
First Author Osama O Zaidat
Title of Work Endovascular Treatment of Acute Ischemic Stroke with the Penumbra System in Routine Practice: COMPLETE Registry Results

Authors must provide written permission/approval from all individuals mentioned by name in the Acknowledgments section of a submitted manuscript. By signing this form, any and all acknowledged persons therefore state that they have read and approved the mention of their names in the Acknowledgment section of the aforementioned paper.

	Name		Signature		Date
Name (1)	Hee Jung Lee	Signature	DocuSigned by: Hee Jung Lee 3845C283C1B8438...	Date	6/3/2021
Name (2)	Nam Nguyen	Signature	DocuSigned by: Nam Nguyen 44B93A13E331494...	Date	6/3/2021
Name (3)	Vincent Ho	Signature	DocuSigned by: Vincent Ho 0C31874C5FF04E9...	Date	6/3/2021
Name (4)		Signature		Date	
Name (5)		Signature		Date	
Name (6)		Signature		Date	
Name (7)		Signature		Date	
Name (8)		Signature		Date	
Name (9)		Signature		Date	
Name (10)		Signature		Date	
Name (11)		Signature		Date	
Name (12)		Signature		Date	
Name (13)		Signature		Date	
Name (14)		Signature		Date	
Name (15)		Signature		Date	
Name (16)		Signature		Date	
Name (17)		Signature		Date	
Name (18)		Signature		Date	
Name (19)		Signature		Date	
Name (20)		Signature		Date	
